# PREVIC: An adaptive parent report measure of expressive vocabulary in children between 3 and 8 years of age

**DOI:** 10.3758/s13428-025-02615-4

**Published:** 2025-02-17

**Authors:** Manuel Bohn, Julia Prein, Jonas Engicht, Daniel Haun, Natalia Gagarina, Tobias Koch

**Affiliations:** 1 Institute of Psychology in Education, Leuphana University Lüneburg, Universitätsallee 1, 21335 Lüneburg, Germany; 2 Department of Comparative Cultural Psychology, Max Planck Institute for Evolutionary Anthropology, Leipzig, Germany; 3 Institute of Psychology, Friedrich-Schiller-University Jena, Jena, Germany; 4 Leibniz-Zentrum Allgemeine Sprachwissenschaft, Berlin, Germany

**Keywords:** Language development, Vocabulary, Individual differences, Item response models

## Abstract

Parent report measures have proven to be a valuable research tool for studying early language development. Caregivers are given a list of words and are asked which of them their child has already used. However, most available measures are not suited for children beyond infancy, come with substantial licensing costs or lack a clear psychometric foundation. Here, we present the PREVIC (Parent Report of Expressive Vocabulary in Children), an open-access, high-quality vocabulary checklist for German-speaking children between 3 and 8 years of age. The PREVIC was constructed leveraging the advantages of item response theory: we designed a large initial item pool of 379 words and collected data from 1190 caregivers of children between 3 and 8 years of age. Based on these data, we computed a range of fit indices for each item (word) and used an automated item selection algorithm to compile a final pool that contains items that (a) vary in difficulty and (b) fit the Rasch (one-parameter logistic) model. The resulting task is highly reliable and shows convergent validity. The IRT-based construction allowed us to design an adaptive version of the task that substantially reduces the task duration while retaining measurement precision. The task – including the adaptive version – was implemented as a website and is freely accessible online (https://ccp-odc.eva.mpg.de/previc-demo/). The PREVIC fills an important gap in the toolkit of researchers interested in language development and provides an ideal starting point for developing converging measures in other languages.

## Introduction

Learning a language is one of the key developmental objectives for children. This learning process is highly variable and leads to persistent individual differences which are related to a wide range of outcome measures later in life (Bleses et al., 2016; Bornstein et al., 2018; Golinkoff et al., 2019; Marchman & Fernald, 2008; Morgan et al., 2015; Pace et al., 2017, 2019; Schoon et al., 2010; Walker et al., 1994). For example, in a longitudinal study spanning 29 years, Schoon et al. (2010) found that relatively poorer language skills at age five were associated with lower levels of mental health at age 34. Given the high predictive validity of early language abilities, researchers and practitioners alike need high-quality, easy-access measures to assess individual differences. However, such measures are rare, and those that exist often come with substantial licensing costs. In this paper, we describe the development of an open, efficient, and valid measure of individual differences in expressive vocabulary.

Child language measures can be broadly categorized into two types: direct and parent report measures. Direct measures of productive and receptive language are generally used with children of 3 years and older. Direct expressive language assessments involve prompting children to generate words or sentences in response to a stimulus, such as a picture or an object. Direct receptive language assessments reverse the logic and require children to match a verbal prompt with a picture or an object. Various direct measures tailored to different languages and age groups have been developed, including measures for English and German (Armon-Lotem et al., 2015; Bohn et al., 2024; Dunn & Dunn, 1965; Dunn et al., 1997; Glück & Glück, 2011; Golinkoff et al., 2017; Kauschke & Siegmüller, 2002; Kiese-Himmel, 2005; Lenhard et al., 2015). Additionally, standardized cognitive ability tests frequently incorporate direct language measures (e.g., Bayley, 2006; Gershon et al., 2013; Wechsler & Kodama, 1949).

Parent report measures, in general, are widely utilized in psychological research. They are particularly popular as screening methods to identify developmental delays (Diamond & Squires, 1993; Pontoppidan et al., 2017). However, it is important to acknowledge that parent reports come with certain caveats, including the potential for selective reporting and social desirability. Consequently, providing a comprehensive assessment of these measures’ overall quality and usefulness is challenging (Morsbach & Prinz, 2006). Nonetheless, some parent report measures have been found to be both reliable and valid (Bodnarchuk & Eaton, 2004; De Cat et al., 2022; Hornman et al., 2013; Ireton & Glascoe, 1995; Macy, 2012; Saudino et al., 1998).

In child language research, parent report measures are often utilized with very young children when direct assessment is challenging. One widely used measure is the MacArthur-Bates Communicative Development Inventories (CDI, Fenson et al., 2007). The CDI asks parents to check those words from a checklist that they believe their child produces and/or understands. This measure has been adapted for a wide range of spoken and signed languages (see Frank et al., 2021 for an overview), with various versions available (e.g., Makransky et al., 2016; Mayor & Mani, 2019), including an online version (DeMayo et al., 2021). Collaborative efforts have facilitated the pooling of CDI data from thousands of children learning different languages into centralized repositories (Frank et al., 2017; Jørgensen et al., 2010). Importantly, the CDI exhibits validity as parental reports align with direct observations and assessments of child language (Bornstein & Haynes, 1998; Dale, 1991; Feldman et al., 2005; Fenson et al., 1994).

However, the use of the CDI – in typically developing children – is limited to 37 months of age. Beyond this point, most children are reported to say all the words on the list. Consequently, there is a need for a comparable measure that can be applied to older children. Even though a wide range of direct language measures exist for preschool and school-aged children, parent report measures can be useful. First, they offer a complementary and perhaps more holistic perspective on children’s language abilities because parents rely on their extensive experience with their children when filling out. Second, they are less dependent on situational factors like children’s fatigue or shyness compared to direct assessments. Finally, they are easier and more economical to apply because they need less time and do not require trained experimenters. This makes them very valuable research and – if normed – screening tools, in particular when dealing with large sample sizes. Existing parent report measures focusing on general cognitive development often include language scales; however, these scales lack detailed information and fail to capture individual differences effectively (Ireton & Glascoe, 1995). For example, the Ages and Stages Questionnaire at 36 months comprises only six items that encompass general communicative behavior, such as whether the child can say their full name when prompted (Squires et al., 2009). One notable example of a dedicated language measure for older children is the Developmental Vocabulary Assessment for Parents (DVAP, Libertus et al., 2015). The DVAP is derived from the words used in the Peabody Picture Vocabulary Test (PPVT, Dunn & Dunn, 1965), a widely used direct measure of receptive vocabulary. As perhaps expected, the DVAP demonstrates high convergent validity, as evidenced by its strong correlation with the PPVT. However, the proprietary nature of the PPVT limits the utility of the DVAP for researchers.^1^ As a consequence, it is unlikely that a comparable “success story” – as observed with the CDI – will emerge where researchers have adapted the original English form to different languages and more efficient forms.

**Fig. 1:**
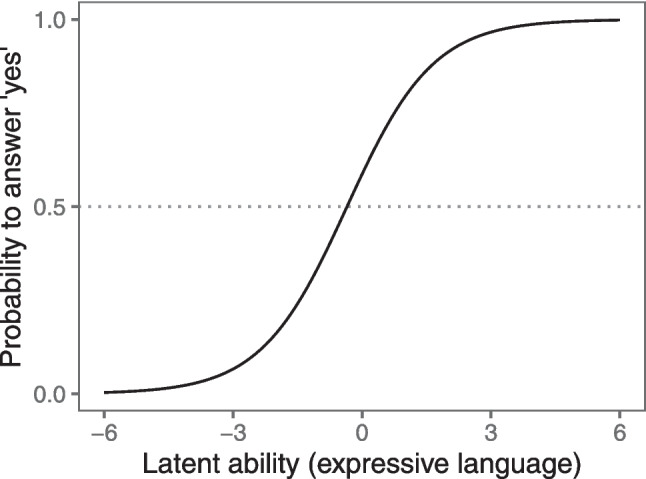
Visualization of the item characteristic curve for a single item according to the Rasch model. The probability of solving an item (*y*-axis) is a direct function of a person’s latent ability (*x*-axis). The curve describing this relationship is known as the item characteristic curve. In the Rasch model, all curves (items) are assumed to have the same slope

A more general issue with existing language measures – including PPVT and DVAP – is a lack of psychometric grounding. Items that make up the scale are selected based on researchers’ intuitions, and there is no clear measurement model that explicates how the different items and test scores are linked to the construct in question (Borsboom, 2006). Item response theory (IRT) offers a theoretical framework to fill this gap and provides a toolkit to develop tasks with a solid psychometric foundation (Kubinger, 2006; Lord, 2012). In unidimensional IRT models, it is assumed that all items measure the same latent construct. Each item is linked to the construct by a probability function (e.g., a logistic curve), which determines how likely a particular response is for individuals with different values on the latent ability (see Fig. 1). The location and shape of this curve is defined by the difficulty of an item (i.e., the value of the latent construct when the probability of solving the item is 50%) and its discrimination (i.e., the slope of the curve showing how the probability to solve the item changes with increasing levels on the latent construct). In a Rasch model, all items are assumed to have equal item discriminations, resulting in parallel item characteristic curves (Rasch, 1980). The great benefit of IRT is that models are testable in that we can quantify the fit of each model and compare competing models. For each item, we can compute fit statistics that indicate how well the model captures the response pattern to the item. Test construction is straightforward in this framework; items are selected that improve the fit to the model.

Besides many other advantages (e.g., objective specificity, sum scores can be used as sufficient statistics), the Rasch model also allows for adaptive testing. All items in a test that conforms to the Rasch model measure the same latent ability and all that differs is their difficulty. As a consequence, the ability of a person can be estimated independently of the particular items that have been completed. This characteristic is leveraged during adaptive testing: individuals do not simply respond to all items in the task but only to the items that are optimally informative given their – constantly updated – individual value on the latent construct. Because all items measure the same latent ability, the resulting scores are nevertheless comparable.

The downside of IRT-based test construction – and probably the reason it is not used more often – is that it requires a larger initial investment (Frey, 2020). To be able to remove items with a poor fit during the selection process requires an initial item pool that is substantially larger than the desired size of the final task. Adaptive testing also needs a large item pool so that there is a sufficient number of items that are optimally informative for different regions of the latent construct. Furthermore, obtaining solid estimates for the item parameters requires large sample sizes. Yet, we believe that these initial costs are clearly outweighed by the benefits that come with IRT-based test construction in the long run.

### The current study

We aimed to develop a high-quality and easy-access vocabulary checklist beyond the CDI for children between 3 and 8 years of age. To ensure the psychometric quality of the task and to allow for adaptive testing, we used IRT to guide item selection and the construction of the item pool. We compiled a large initial pool of candidate items. Next, we collected a large data set and analyzed it using the simplest version of an IRT model, the Rasch model (Rasch, 1980). The main reason behind this fairly restrictive approach was that only when the Rasch model holds is the number of solved items (sum score) a sufficient statistic and can be used to represent an individual’s value on the latent construct (Birnbaum, 1986). Based on the first analysis, we computed a range of item-level indices that captured how difficult the item was and how well it fit the Rasch model. We then used an automated procedure to construct a smaller pool of items with varying difficulties that all fit the Rasch model. Finally, we report the results of two studies assessing the convergent validity of the task. To ensure easy access, we implemented the checklist as an interactive web app. Furthermore, the task, the item pool, and all associated materials are openly available for other researchers to use.

## Methods

### Task design and implementation

**Fig. 2:**
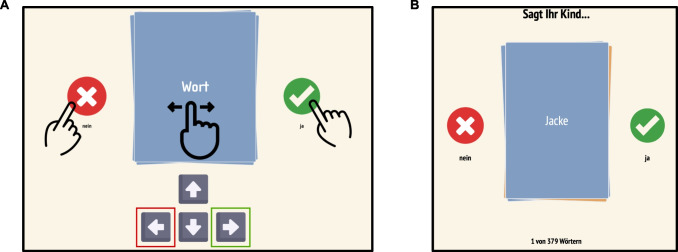
Task implementation. **A** Instructions provided to parents demonstrating the functionality of the task. The word was presented on a card in the middle of the screen. Parents could indicate whether or not their child says a word by swiping the card left (no) or right (yes), touching or clicking the “yes” or “no” symbol or pressing the left or right arrow key on the keyboard. **B** Screenshot from the task presenting the German word Jacke (en: jacket)

We decided to use an interactive format instead of presenting parents with a long list of words in order to increase the number of items while keeping the task engaging. The task was implemented as a web app using html and JavaScript and ran in every modern web browser on computers, tablets, and smartphones. Words were presented one by one, and caregivers could indicate whether or not their child said a word either by using the familiar swipe-left/swipe-right functionality, by clicking symbols or using arrow keys on a keyboard (see Fig. 2A). For example, caregivers saw the word “Jacke” (en: jacket) on a card (color-coded by part of speech: noun = blue, adjective = orange, verb = green) in the center of the screen; to report that their child says the word, they would swipe the card to the right side of the screen which would make the card go away and the next in the deck appear. We included a lightweight back-end that registered the last completed trial so that caregivers could take breaks and even switch devices during the task. There was no time limit for the completion of the task.

For each child, we created a personalized link that connected the caregiver’s responses to the child’s entry in our database. After clicking the link, participants saw a short video in which the first author introduced the rationale of the study. Next, they were introduced to the functionality of the task (Fig. 2A) and how to respond. We used the same instructions for how to judge whether a child says a word or not as the German version of the CDI (FRAKIS, Szagun et al., 2009).

### Item pool generation

**Fig. 3:**
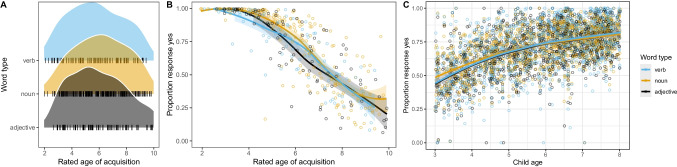
Initial item pool. **A** Distribution of items across word types and rated age-of-acquisition. **B** Item-based association between rated age-of-acquisition and caregiver responses by word type averaged across participants. **C** Child-based association between age and caregiver responses by word type averaged across items

We aimed to create an item pool with items of different word classes and varying semantical difficulty. We used age-of-acquisition (AoA) ratings as a rough indicator of anticipated item difficulty. Previous work has shown strong associations between AoA ratings and how likely children are to know a word (Bohn et al., 2021, 2024). We started the process by compiling a list with AoA ratings for 3921 German words from various sources (Birchenough et al., 2017; Łuniewska et al., 2019; Schröder et al., 2012). We excluded words with AoA ratings above ten. The remaining words were ordered by rated AoA and then split into ten lists with 344 words each. A research assistant with a background in linguistics went through the lists and selected words that (a) were indicative of language abilities more broadly (avoiding very specialized terms) and (b) that were different from one another in that they were not highly semantically related (to avoid words that are learned in the same context). For each list, we aimed for roughly 35 words from the three word classes; 17 nouns, nine verbs and nine adjectives. The so-generated item pool had 379 words, of which 197 were nouns, 92 were verbs, and 90 were adjectives. Figure 3A shows how the items were distributed across AoA ratings and word types.

### Data collection

Ext, we aimed to collect data for all 379 items from a large sample of parents with children between 3 and 8 years of age. We aimed to have at least 100 complete responses per year (e.g., 100 parents with children between 3.0 and 4.0). This data would then be used to estimate item parameters to be used during the selection process.

### Participants

Participants were recruited via a database of children whose caregivers indicated an interest in participating in studies on child development and who additionally signed up for online studies. All children lived in Leipzig, Germany, an urban Central-European city with approximately 600,000 inhabitants. The city-wide median individual monthly net income in 2021 was ~ 1600€. Children growing up in Leipzig mostly live in nuclear two-generational families. Socioeconomic status was not formally recorded, although the majority of families in the database come from mid to high socioeconomic backgrounds with high levels of parental education. In addition, it is very likely that the online format caused selective responding and skewed the sample towards highly motivated and interested families. Caregivers received an email with a personalized link to the study. Approximately 1 week after the first email, they received a reminder if they had not yet finished the study. We contacted caregivers of 4094 children; caregivers of 1826 children started the study of which 1190 (29.00%) completed all 379 items. All subsequent analyses are based only on the complete data. Table 1 shows the age and sex distribution of participants.

**Table 1:** Participants per age group and sex

Age group	*N*	Female
3–4 years	176	82
4–5 years	191	84
5–6 years	221	113
6–7 years	291	142
7–8 years	308	148
> 8 years	3	1

*Note.* Children in the > 8 years group were very close to 8; see Fig. 2C

#### Descriptive results

Figure 3 visualizes the results. On an item level, we saw strong negative correlations between caregiver’s responses and rated ages of acquisition. The less likely caregiver’s were to say their child says a word, the higher the rated AoA. This relation was the same for nouns, verbs, and adjectives. On a child level, we saw that the older children were, the more words they used according to their caregivers. These results reflect highly expected patterns and served as a sanity check for the design and implementation of the task.

### Item selection

The item selection procedure aimed to generate an item pool with items that fit the Rasch model. We selected items in three steps. In the first step, we excluded items using conventional cut-offs for indices that quantify the fit of each item to the Rasch model. The goal was to remove a large number of items with a poor fit to reduce the computational burden in subsequent steps. In step 2, we used an automated item selection procedure to select an optimal subset of items from the remaining pool. We focused on the fit of the Rasch model as well as variation in item difficulty (to measure in different regions of the latent dimension). Finally, in step 3, we submitted the items selected in step 2 to an analysis of differential item functioning (DIF).

IRT models were implemented in a Bayesian framework in R using the *brms* package (Bürkner 2017, 2019) unless otherwise stated. We predicted the probability of a correct answer based on a participant’s latent ability and item characteristics. We fit two classes of models: Rasch and Birnbaum (2PL) models. The main difference between these two models lies in their assumption about how the probability of solving an item changes with ability levels (item discrimination). Here, the Rasch model assumes that the rate of change (i.e., the slope of the logistic curve) is the same for all items, while the 2PL model allows item discrimination parameters to vary between items.

#### Step 1: Fit-based item selection

**Fig. 4:**
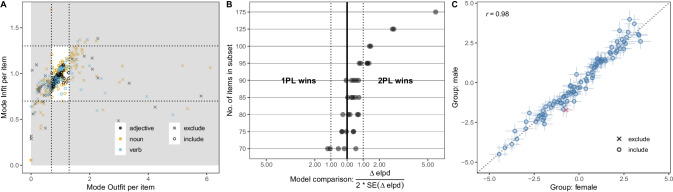
Three steps of item selection. **A** In- and Outfit values for all 379 items in the initial item pool. Items that fell into the grey region (*crosses*) were excluded. The color shows the different word types. *Dashed lines* show cut-off values of 0.7 and 1.3. **B** Model comparison ratio comparing the fit of a Rasch model to the fit of a 2PL model for different numbers of items (*y*-axis). Each *point* shows an independent run of the item selection procedure and subsequent model comparison (five per subset). The *x*-axis title shows how the ratio is computed. Values left of 0 indicate a better fit of the Rasch model, and values to the right are a better fit of the 2PL model. The *dashed line* marks a ratio of 1, which we assumed to be the point when one of the models clearly provided a better fit. **C** Correlation between item parameters estimated separately by sex. *Points* show the mode of the posterior distribution for each item with 95% CrIs. *Point color* and *shape* denote items that were excluded

In- and Outfit quantify the deviation of a person’s response to an item from what the model predicts, based on the difficulty of an item and the person’s ability parameter. That is, both indices are a direct measure of how well the model was able to predict the responses to an item. We fit a Rasch model to the data and computed In- and Outfit values based on draws from the model’s expected posterior predictive distribution (using the function add_epred_draws) for each item and person combination. For each draw, we computed the residual between predicted and observed responses. The mean of the squared residuals is the Outfit; to obtain the Infit, the mean squared residuals are weighted by item information (i.e., the probability of responding yes multiplied by the probability of answering no). The result was a distribution of values for each item and index. For each item, we then computed the mode for each index. The closer to 1 the index is, the better the fit. Figure 4 visualizes the results. We used the cut-off values suggested in the literature (Bond & Fox, 2013; Debelak et al., 2022) and excluded items with In- or Outfit values below 0.7 and above 1.3. Like all heuristics, these cut-offs are, to some extent, arbitrary. Yet, in the present context, they served the purpose of removing a large number of potentially unsuitable items. This procedure led us to exclude 167 of the 379 items in the pool, leaving 212 for the automated item selection.

#### Step 2: Automated item selection.

The goal of this step was to select items with different levels of difficulty that fit the Rasch model. Selecting items based on these criteria ensured that (a) the final item pool allowed for precise measurement in different regions of the latent ability and (b) the number of solved items is a sufficient statistic for an individual’s ability. Such an item pool is then optimally suited for adaptive testing because items differ in difficulty but measure the same latent dimension. This way, individuals with different ability levels can be shown different items while still ensuring that the eventual scores are directly comparable.

First, we defined an objective function that reflected the selection criteria, which would later be used in the automated selection process. Items should vary in their difficulty but still cover all sections of the latent ability; we quantified this requirement as the standard deviation of the distance (in difficulty estimates) between adjacent items. The distance between adjacent items was computed by first sorting all items in the subset by difficulty and then subtracting the difficulty of adjacent items. A lower standard deviation of these distances indicates little variation between them and thus an overall more equal spacing. Items should also fit the Rasch model; we quantified this requirement in three ways. First and second, we used the In- and Outfit values for each item computed in the previous step. Third, we computed modification indices for each item. For this, we re-fitted the Rasch model using the package *lavaan* (Rosseel, 2012) and used the function *modindices* to obtain modification indices. Broadly speaking, modification indices quantify the improvement in model fit (in terms of the chi-square test statistic) when an item is dropped (Rosseel, 2012).

The objective function was the sum of these four components. Before summation, we multiplied the different components by constants to bring them on a comparable scale and to emphasize certain components over others: the standard deviation for item difficulties was multiplied by – 1/3, Infit values by – 4, Outfit values by – 2 and modification indices by – 1/100. The resulting score was always negative, so that larger individual values led to more negative values. Because the process described below aims to maximize the score, this meant minimizing the individual values.

Following Bohn et al. (2024), we employed simulated annealing (Kirkpatrick et al., 1983) as a method to identify the most optimal items for any given subset size. The process systematically explores the vast space of possible subsets, commencing from a randomly selected initial subset. Subsequently, small random changes are proposed by exchanging some items within the subset under consideration with others located outside it. If a proposed change leads to an improvement in the objective function’s value, the proposal is accepted, and the enhanced subset becomes the starting point for subsequent proposals.

To prevent the process from becoming trapped in local optima, it probabilistically accepts proposals that decrease the value of the objective function. The probability of accepting a proposal that reduces the objective function is influenced by a parameter known as “temperature,” which gradually decreases from an initially high value to a lower value during the simulation. In the early “hot” phase, the process explores the search space more freely, accepting decreasing proposals often enough to enable movement between local optima separated by less effective subsets, facilitating the discovery of global optima. As the simulation progresses into the later “cool” phases, the process converges towards a more focused “hill climbing” search, where only increasing proposals are accepted. This fine-tunes the best subset discovered during the hot phase, resulting in a more refined and optimized solution.

The simulated annealing algorithm finds the optimal items for a given size of the subset but does not answer the question of what the optimal size is. To answer this question, we applied the algorithm to subsets of different sizes. Our goal was to find the largest subset for which the Rasch model provided a good fit. For each size, we therefore compared the fit of a Rasch model to a 2PL model using Bayesian approximate leave-one-out cross-validation (Vehtari et al., 2017) based on differences in expected log posterior density (ELPD) estimates and the associated standard error (SE). Based on suggestions in the literature (Sivula et al., 2020), we considered models to be equivalent up to a point when the ELPD in favor of a model exceeded two times the standard error of the difference.

Figure 4B visualizes the model comparison and shows that the Rasch model provided a good fit for subsets up to 90 items. We therefore decided on 90 items as the size of the final item pool. We ran the simulated annealing algorithm 20 times and selected the 90 items that were returned most often (the same 86 items were returned on every run).

#### Step 3: Differential item functioning

The final step of item selection assessed differential item functioning (DIF, see Bürkner, 2019). DIF describes a situation when items show differential characteristics for subgroups that otherwise have the same overall score (Holland & Wainer, 2012). We assessed DIF based on sex (male and female). We estimated separate item parameters for the two groups and assessed whether their 95% CrI overlapped. Figure 4C shows that the item parameters were very similar in the two subgroups. However, one item (“verloben”, en: to get engaged) had to be excluded. Thus, the size of the final item pool was 89 items, 43 (48%) of which were nouns, 20 (22%) were verbs, and 26 (29%) were adjectives.

### Adaptive testing

The large and diverse item pool allowed us to create an adaptive version of the PREVIC in addition to the complete checklist. The general idea of an adaptive test is to only show the caregiver the most informative items given the (continuously updated) individual ability. As a consequence, items that are too easy or too difficult are omitted and the test becomes substantially shorter while retaining the same level of measurement precision.

In order to determine the most informative items, the ability of the child has to be estimated during the test. To achieve this, we implemented a maximum likelihood estimator in html and TypeScript (which is compiled to native JavaScript). As a consequence, the adaptive version is still fully portable and can be run in any modern web browser. The estimated ability “is the ability value that maximizes the likelihood function $L\left(\theta \right)$” (Magis & Raîche, 2012), given the item response ${y}_{i}$ (either 0 or 1) and the item difficulty ${\alpha }_{i}$ (Eid & Schmidt, 2014):

$$L\left(\theta \right)=\prod_{i=1}^{p}\frac{{\text{exp}}^{{y}_{i}*\left(\theta -{\alpha }_{i}\right)}}{1+{\text{exp}}^{\theta -{\alpha }_{i}}}$$

The maximum likelihood estimation is implemented using a line search algorithm that converges when the maximum of the likelihood distribution has been reached. Based on the estimated ability, the task will then select the next item from the pool so that the difficulty is nearest to the current ability level (Urry, 1970). This procedure is equivalent to selecting items with the maximum information criterion when using a Rasch model (Magis & Raîche, 2012).

At the beginning of the test, the ability level is set to 0. A person-specific ability estimation is not yet possible using maximum likelihood estimation after the first item, and we therefore followed the convention to set the ability level to – 10 if the answer was “no” and 10 if the answer was “yes” (e.g., implemented in the R package catR, Magis & Barrada, 2017). The test then continues until a pre-specified level of measurement precision (standard error of the ability estimate) is reached or until all items have been used. Users can set the desired level of measurement precision at the beginning of the test (e.g., SE of 0.3, 0.4 or 0.5), which again influences its length (larger SE means shorter test). In the end, the user downloads a file containing the following information: the estimate of the latent ability of the participant (on the same scale as item difficulties), the SE of the ability estimate (also used to terminate the test), the an the final ability level, the answered items (including the word itself and its difficulty) and the participant’s response pattern.

We validated the implementation of our estimator by comparing its ability estimates and selected items to those of the catR package in a number of simulations. The results were identical and only differed beyond the fifth decimal because JavaScript and R differ in their implemented floating-point number format. The code to run the simulations can be found in the associated online repository.

### Psychometric properties

**Fig. 5:**
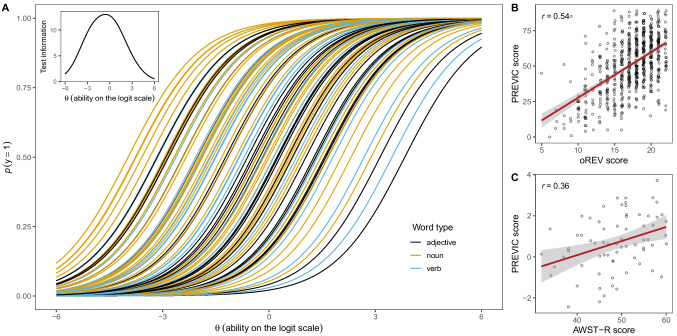
Item characteristics and validity. **A** Item characteristic curves for the 89 items colored by word type. The* inset on the upper left* shows the test information curve. **B** Correlation between PREVIC and oREV scores. *Points* show aggregated scores of individuals in the two tasks. Points have minimal horizontal noise added to avoid overplotting. The *red line* shows a regression line (with 95% CI) based on a linear model. **C** Posterior model estimates for oREV scores and age (scaled) in a model predicting PREVIC scores. *Points* show posterior means with 95% CrI

The final item pool consisted of 89 items of varying difficulty that fit the Rasch model (see Fig. 5A). Next, we investigated the reliability and convergent validity of a task including the full item pool as well as the adaptive version.

#### Reliability

We computed KR-20 (Kuder & Richardson, 1937) and Andrich Reliability (Andrich, 1982). Both indices indicated excellent reliability (KR-20 = 0.97; Andrich = 0.97).

#### Convergent validity

We assessed convergent validity in two studies. First, we compared PREVIC scores to a direct assessment of children’s receptive vocabulary using the oREV (Bohn et al., 2024). The oREV asks children to select a picture (out of four) upon hearing a word. It has 22 items that fit the Rasch model. Because the oREV is also available as a web application, we sent out emails to all caregivers who provided complete data in the data collection that led to the construction of the PREVIC (*N* = 1190) and asked them to have their child complete the oREV. We obtained oREV data from 692 children (337 female, ${m}_{\text{age}}$ = 5.78, range = 3.02–8.00) which corresponds to a response rate of ~ 58%. We found a substantial correlation between caregiver’s answers to questions about their children’s expressive vocabulary in the PREVIC and a direct assessment of children’s receptive vocabulary in the oREV (*r* = 0.54; 95% CI = 0.48–0.59; Fig. 5B).

Second, we directly assessed children’s productive vocabulary using the AWST-R (Aktiver Wortschatztest für 3- bis 5-jährige Kinder – Revision, Kiese-Himmel, 2005). The AWST-R is unavailable as an online version, and we resorted to in-person testing. Children were tested in a separate room in a child laboratory by a trained experimenter while parents filled out the adaptive version of the PREVIC on a tablet in the waiting room. We used an SE of 0.4 for the ability estimate as criterion for termination of the adaptive PREVIC. A total of 70 children and their parents participated in the study (38 female, ${m}_{\text{age}}$ = 5.02, range = 4.12–5.94). We found a substantial correlation between PREVIC and AWST-R scores (*r* = 0.36; 95% CI = 0.13–0.55; Fig. 5C). This correlation was lower compared to the oREV even though the AWST-R is – like the PREVIC – a measure of expressive vocabulary. We discuss this result in more detail below. Nevertheless, the results of these two studies speak to the convergent validity of the PREVIC.

## Discussion

This paper describes the construction and validation of the PREVIC, an adaptive parent report measure of productive vocabulary in German-speaking children between 3 and 8 years of age. Following the logic of widely used vocabulary checklists for younger children (Fenson et al., 2007), the PREVIC presents caregivers with individual words and asks if the child speaks these words. The items (words) that make up the PREVIC were selected using item response theory: we started with a large initial item pool of 379 words, from which we selected 89 items that fit the Rasch model. The resulting highly reliable task shows convergent validity when contrasted with a direct receptive vocabulary measure. Leveraging IRT allowed us to devise an adaptive version of the task in which only the most informative items are presented. The task is implemented as a web app and can be used with any device that runs a modern web browser. The task itself (adaptive and complete checklist) as well as the source code are freely available online.

The PREVIC fills an important gap in the tool kit of researchers studying language development beyond infancy, particularly during the preschool years. It complements direct assessments of children’s vocabulary by providing an additional perspective on children’s vocabulary skills. Parents observe children for extended periods of time and their assessment therefore provides a more aggregated measure. Parental reports are also immune to momentary fluctuations in children’s motivation and attention that might influence the results of direct assessments. Nevertheless, parental reports remain indirect measures and are ideally combined with direct assessments whenever feasible. Given that the PREVIC is short – in particular the adaptive version rarely takes more than 5 min to complete – it can easily be filled out by parents during a lab or any other institutional, e.g., pediatrician, visit. Its implementation as a web app even allows for sending it to families before or after a visit.

At present, the PREVIC is available only in German. However, with inclusivity and broad applicability in mind, we have made the entire source code available. This not only facilitates its adaptation to other languages and allows researchers to use the same user interface. Encouragingly, preliminary feedback indicates that parents find the interface intuitive and user-friendly. The CDI has seen expansive adaptation across various languages (see Frank et al., 2021 for a summary). Such adaptations are usually not complete translations in that some words are removed and others added to capture the linguistic nuances and specificities of each language. However, Łuniewska et al. (2019) found that the order of acquisition (and thus presumably the difficulty) was similar for many words across seven languages. Hence, most items in the current pool could be translated and re-used if the PREVIC were to be adapted to different languages. Nevertheless, a comprehensive reassessment of item properties would be highly desirable. For adaptive testing, it would even be mandatory. Taken together, we hope that our commitment to openness will put the PREVIC on a similar trajectory as the CDI.

## Limitations

The sample we tested was not a representative sample: It only contained families living in Leipzig, Germany, who volunteered to participate in research on child development *and* who additionally indicated that they were interested in participating in online studies. These multiple steps of (self-)selection most likely skewed the sample to more affluent and educated parents, though we have no demographic data to assess this claim. We think the most likely consequence is that the variation in our sample was reduced compared to the general population and that the probability of knowing a particular word would be somewhat lower in a representative sample. The data we collected during the construction of the PREVIC should therefore not be seen as a normative data set. Instead, the PREVIC is first and foremost a research tool that can be used to measure variation in receptive vocabulary in a given sample.

When assessing convergent validity, we found a somewhat lower correlation between PREVIC scores and a direct measure of children’s expressive vocabulary (AWST-R) compared to receptive vocabulary (oREV). Potential reasons for this pattern could be as follows: the AWST-R has not been revised in nearly 20 years, and some of its images may now seem outdated (e.g., “rauchen” (Eng. “smoking”) or “telefonieren” (Eng. “to phone”)), making them less suited to assess expressive everyday language. Relatedly, mean performance in the validation study was near the upper end of the AWST-R score range (though not at ceiling), which made it challenging to discriminate between individuals due to the scarcity of very difficult items. Another possible reason is the use of a relatively large standard error (0.4) as the criterion for terminating the PREVIC. On average, parents responded to only around 34 items (out of 89), often completing them in under five minutes. A smaller standard error of, e.g., 0.3 would have resulted in a longer test (more items) and a more precise estimate of the person’s ability. In sum, these factors may have led to less precise measurement at both ends of the scale, potentially contributing to the relatively lower correlation. We therefore recommend to use a smaller standard error of e.g., 0.3 for the adaptive version. Note, however, that a standard error of 0.2 usually leads to a presentation of all items in the pool.

Many points of criticism that apply to parental report measures apply to the PREVIC as well. Parents might be biased in their assessment and more recent events might have a stronger influence on their responses compared to more distant ones. Furthermore, compared to measures for younger children, the PREVIC might be less accurate in an absolute sense because children speak much more words and parents spend less time with their children as they get older. Nevertheless, we found a substantial correlation with a direct measure, suggesting that the PREVIC accurately captures relative individual differences.

## Conclusion

We designed the PREVIC with a commitment to psychometric rigor; its grounding in item response theory provides a clear measurement model and specifies how individual items relate to each other and the underlying psychological ability. This approach not only strengthens the PREVIC’s validity in assessing receptive vocabulary but also serves as a methodological reference for developing tests in other areas. By making the PREVIC openly accessible, we actively contribute to the collective resource pool for researchers in language development, ensuring they have another reliable tool at their disposal.

## Data Availability

The data sets generated during and/or analyzed during the current study are available in the following repository: https://github.com/manuelbohn/previc/. Materials used in the task can be found in the following repository: https://ccp-odc.eva.mpg.de/previc-demo/.

## Footnotes

1 When the first author approached the license holder of the PPVT in Germany to ask if we could use the German version of the PPVT to build a parental report measure, we were told that we would have to pay for every administration of the new measure and we would not be allowed to openly share the materials.

## Code availability

The task can be accessed via the following website: https://ccp-odc.eva.mpg.de/previc-demo/. The corresponding source code can be found in the following repository: https://github.com/ccp-eva/previc-demo.
